# Application of Magnetic Nanoparticles to Gene Delivery

**DOI:** 10.3390/ijms12063705

**Published:** 2011-06-07

**Authors:** Daisuke Kami, Shogo Takeda, Yoko Itakura, Satoshi Gojo, Masatoshi Watanabe, Masashi Toyoda

**Affiliations:** 1 Research Team for Vascular Medicine, Tokyo Metropolitan Institute of Gerontology, 35-2 Sakae-cho, Itabashi-ku, Tokyo 173-0015, Japan; E-Mails: yitakura@tmig.or.jp (Y.I.); satoshigojo-tky@umin.ac.jp (S.G.); mtoyoda@tmig.or.jp (M.T.); 2 Laboratory for Medical Engineering, Division of Materials and Chemical Engineering, Yokohama National University, 79-1 Tokiwadai, Hodogaya-ku, Yokohama 240-8501, Japan; E-Mails: smilesnow1987@gmail.com (S.T.); mawata@ynu.ac.jp (M.W.)

**Keywords:** magnetic nanoparticles, Magnetofection, gene delivery, polyethylenimine

## Abstract

Nanoparticle technology is being incorporated into many areas of molecular science and biomedicine. Because nanoparticles are small enough to enter almost all areas of the body, including the circulatory system and cells, they have been and continue to be exploited for basic biomedical research as well as clinical diagnostic and therapeutic applications. For example, nanoparticles hold great promise for enabling gene therapy to reach its full potential by facilitating targeted delivery of DNA into tissues and cells. Substantial progress has been made in binding DNA to nanoparticles and controlling the behavior of these complexes. In this article, we review research on binding DNAs to nanoparticles as well as our latest study on non-viral gene delivery using polyethylenimine-coated magnetic nanoparticles.

## 1. Introduction

Nanotechnology describes the creation and utilization of materials, devices, and systems through the control of nanometer-sized materials and their application to physics, chemistry, biology, engineering, materials science, medicine, and other endeavors. In particular, intensive efforts are in progress to develop nanomaterials for medical use as agents that can be targeted to specific organs, tissues, and cells. For example, magnetic nanoparticles (MNPs) are being used clinically as contrast agents for magnetic resonance imaging (MRI) (Table 1). MRI is a noninvasive technique that can provide real-time high-resolution soft tissue information [1,2]. MRI image quality can be further improved by utilizing contrast agents that alter proton relaxation rates [3–8]. MNP-based drug delivery systems (DDS) [9–11], and treatments of hyperthermia [12–21], using MNPs have been studied for over a decade. Furthermore, researchers have reported that MNPs have been useful in hyperthermic treatment for various cancers *in vivo* [22–31]. Nanotechnology-based anti-cancer agent DDS have already been approved, such as pegylated liposomal doxorubicin (DOXIL) for ovarian cancer [32–37]. MNPs have been used effectively as transfection reagents for introducing nucleic acids (plasmids or siRNAs) [38–53], or viruses (retrovirus, or adenovirus) [44,54–56] into cells. Our own research is focused on MNP-mediated gene delivery systems (called as “Magnetofection”).

## 2. Gene Delivery

**Figure 1:**
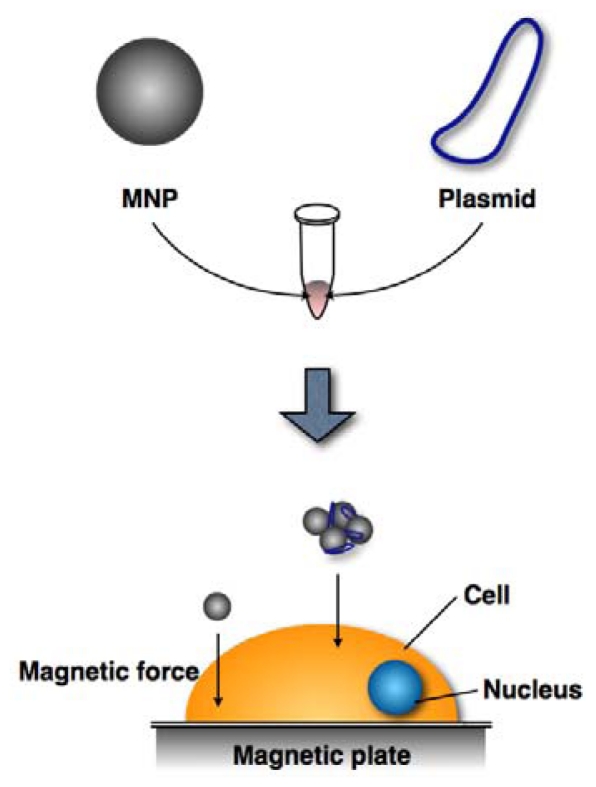
MNP gene delivery system (Magnetofection). Plasmids are bound to MNPs, which then move from the media to the cell surface by applying a magnetic force.

Gene delivery techniques efficiently introduce a gene of interest in order to express its encoded protein in a suitable host or host cell. Currently, there are three primary gene delivery systems that employ viral vectors (retroviruses and adenoviruses), nucleic acid electroporation, and nucleic acid transfection. These systems vary in efficacy (Table 2). Gene delivery by viral vectors can be highly efficient (80–90%) but may insert viral vector nucleic acid sequences into the host genome, potentially causing unwelcome effects, such as inappropriate expression of deleterious genes. Electroporation is also a highly efficient technique for introducing foreign genes into a host (50–70%); however, half of the recipient cells die due to the electrical stimulation. Transfection reagents do not efficiently deliver nucleic acids into cells (20–30%); however, cell viability is largely preserved and the method is safe enough for clinical use. Therefore, this method holds relatively more promise for medical applications, provided that its efficiency can be improved. MNPs are already in use by basic researchers to increase transfection efficiencies of cultured cells. Thus, MNP-nucleic acid complexes are added to cell culture media and then onto the cell surface by applying a magnetic force (Figure 1).

Oxide nanoparticles mixed with high magnetic moment compounds such as CoFe_2_O_4_, NiFe_2_O_4_, and MnFe_2_O_4_ exhibit superior performance compared to other magnetic materials [62,63]. However, these nanoparticles are highly toxic to cells, limiting their use for *in vivo*, and *in vitro* biomedical applications [64–67]. However, iron oxides such as magnetite (Fe_3_O_4_) and maghemite (γ-Fe_2_O_3_), in particular, possess high magnetic moments, are relatively safe, and currently in clinical use as MRI contrast agents [57–61]. These iron oxide based-magnetic materials are also suitable for biomedical applications. Fe^3+^ is widely dispersed in the human body so leaching of this metal ion from nanoparticles should not reach toxic concentrations [68,69]. As a result, maghemite is a popular choice for MNPs used biomedical applications. It is very important to modify the surface of MNPs so that they can be used for biomedical applications. Thus, MNPs are coated with compounds such as natural polymers (proteins and carbohydrates) [70–75], synthetic organic polymers (polyethylene glycol), polyvinyl alcohol, poly-l-lactic acid) [72,76–78], silica [79], and gold [80,81]. These surface coating agents prevent nanoparticle agglomeration, cytotoxicity, and add functionality. MNPs agglomerate readily in aqueous solutions around pH 7 [82], and it is difficult to control the properties and amounts of agglomerated MNPs. The greater toxicity of MNPs compared to those of microparticles can be attributed to their high surface to volume ratio [83]. Coating agents prevent the leaching of potentially toxic components from MNPs. In fact, the cytotoxicity of uncoated NiFeO_4_ MNPs is dramatically decreased by coating with cationic polymer, polyethylenimine (PEI) [84–86]. PEI, a cationic polymer, is widely used for nucleic acid transfection [87–89] and also serves as a nanoparticle dispersant [90]. PEI-coated MNPs enhance transfection efficiency [38,41,42,44–46,48,49,51,54,55].

## 3. Cell Transplantation Therapy Using MNPs

Autologous cell transplantation has been widely used in the clinic for decades. Delivering therapeutic genes to patients using their own cells avoids using immunosuppressive drugs. We reasoned, therefore, that a non-viral gene delivery system using iron oxide-based MNPs could provide a powerful tool for next-generation therapies. Gene delivery using MNPs has been successful for delivering nucleic acids into living cells with high efficiency and low cytotoxicity [38,41,42,44–46,48,49,51,54,55]. Currently, there are several methods for inducing cellular differentiation.

**Figure 2:**
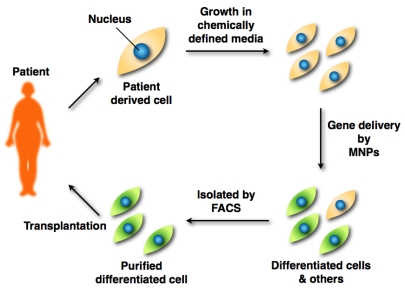
Strategy for cell transplantation therapy. A patient’s cells are cultured in chemically defined media. MNP-transfected cells by the introduced gene are isolated by FACS. FACS-purified differentiated cells are transplanted into the patient.

One of these methods, termed direct reprogramming, or direct conversion, has successfully yielded induced cardiomyocytes, induced neurons, reprogrammed pancreatic β cells, and induced pluripotent stem cells (iPSCs) [91–95]. Direct reprogramming represents a more straightforward strategy to treat diseases involving loss of function by specific cell populations compared to approaches requiring an intermediate embryonic stem cell. Thus, patient-derived differentiated cells by gene transfer are suitable for autologous cell transplantation, potentially resulting in faster patient recoveries. The scheme is classified into *ex vivo* gene therapy. The steps involved in this technique are as follows: (1) Patient-derived cells (such as fibroblasts) are cultured in chemically defined media *in vitro*; (2) These cells are transfected by MNPs, and differentiated into functional cells; (3) Differentiated cells are isolated by fluorescence-activated cell sorting (FACS); (4) FACS-purified differentiated cells are transplanted into the patient’s target tissue (Figure 2).

Here we briefly describe the magnetofection [96], and our latest study concerning non-viral gene delivery using deacylated polyethylenimine coated MNPs.

## 4. Gene Delivery Using MNPs and Magnetic Force

**Figure 3:**
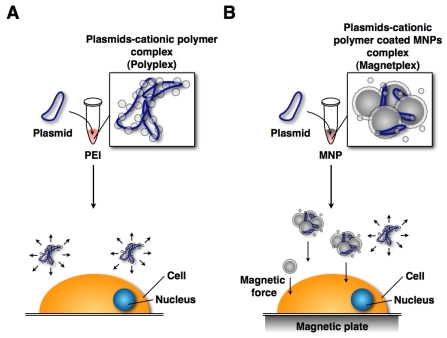
Gene delivery systems using a transfection reagent (cationic polymer) and MNPs: (**A**) Gene delivery system using transfection reagent. The polyplex moves randomly in culture medium; (**B**) Magnetofection system. The magnetoplex only moves to the cell surface.

The mechanism of magnetofection is similar to using transfection reagents (Lipofectamine 2000, FuGENE HD, and PEI). The only difference is that the plasmids form complexes with cationic polymer-coated MNPs (called as “Magnetoplex”) [42,48,97–99] (Figure 3). Figure 3 shows the two difference techniques. The behavior of magnetoplex is readily controlled by magnetic force. Upon binding to the cell surface they are taken up by endocytosis [51,100,101]. Thus, the transfection efficiency was increased.

Many researchers have described magnetofection methods (Table 3). They modified the surface of iron oxide-based MNPs to increase transfection efficiency and reduce cytotoxicity. To achieve this, some investigators selected coating agents such as anionic surfactants (oleic acid, lauroyl sarcosinate) [42,50,102], a non-ionic water-soluble surfactant (Pluronic F-127) [42], fluorinated surfactant (lithium 3-[2-(perfluoroalkyl) ethylthio]propionate) [54], a polymer (polyethylene glycol, poly-l-lysine, poly(propyleneimine) dendrimers) [40,103,104], carbohydrates (Chitosan, Heparan sulfate) [41,47], silica particles (MCM48) [49], proteins (serum albumin, streptavidin) [40,55], hydroxyapatite [105], phospholipids [49,50], a cationic cell penetrating peptide (TAT peptide) [43], non-activated virus envelope (HVJ-E) [47], a transfection reagent (Lipofectamine 2000) [53], and viruses (adenovirus, retrovirus) [44,54–56]. These coating agents are often used in conjunction with PEI. PEI is a well-known cationic gene carrier with high transfection efficiency. However, the high toxicity, depended on its molecular weight, has limited its use as a potential gene carrier. Thus, the PEI was modified to increase transfection efficiency, and decrease cytotoxicity [88,106]. To enhance transfection efficiency, most researchers used the PEI, or the modified PEI to coat the nanoparticle surface [38,41,42,44–46,48,49,51,54,55,102,107]. PEI-coated MNPs are stable in water, bind nucleic acids, and control MNP behavior by magnetic force. In addition, linear PEI possesses low cytotoxicity compared with branched PEI *in vivo* and *in vitro* [108,109] The highest transfection efficiencies have been achieved using 25,000 molecular weight linear PEI [89]. However, PEI cytotoxicity due to its acyl groups has been described [88]. Therefore, our group focused on commercial deacylated PEI (Polyethylenimine “Max” (PEI “Max”), Polysciences Inc.) as an MNP (γ-Fe_2_O_3_, *d* = 70 nm, CIK NanoTek) coating agent.

**Figure 4:**
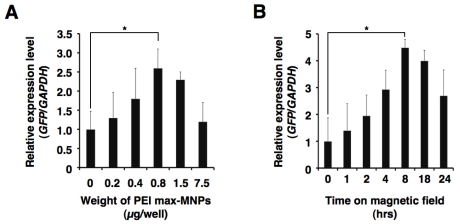
Optimum conditions for PEI max-MNPs magnetofection. To optimize conditions, we varied volume (**A**) and time on the magnetic plate (**B**). These results were evaluated by quantitative real-time RT-PCR. The relative expression level (*GFP*/*GAPDH*) in the human fetal lung-derived fibroblasts (TIG-1 cells) treated with PEI max alone (A), and in the absence of magnetic force (0 h) (B) was defined as 1. Optimal transfection conditions were established when TIG-1 cells were treated with 0.8 μg PEI max-MNPs and 2.0 μg pCAG-GFP for 8 h on the magnetic plate in either a six-well plate or a 35 mm dish. The asterisk (*) indicates a significant difference (*P* < 0.05).

**Figure 5:**
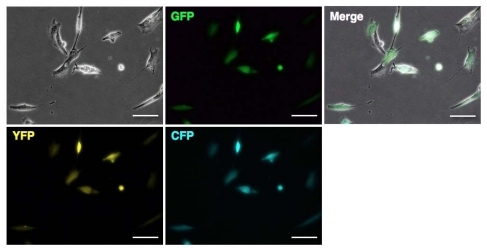
Transfection of TIG-1 cells with multiple genes using PEI max-MNPs. TIG-1 cells were simultaneously transfected with GFP, CFP, and YFP expression vector plasmids. TIG-1 cells were treated with 0.8 μg of PEI max-MNPs and 0.7 μg each of pCAG-GFP (GFP, provided by Dr. Nishino), pPhi-Yellow-N (YFP, Evrogen), and pAmCyan1-C1 (CFP, Clonetech) for 8 h on the magnetic plate in a six-well plate or a 35 mm dish. White bar indicates 200 μm.

Deacylated polyethylenimine (linear, 25,000 molecular weight) is built from the same polymer backbone as the popular linear polyethylenimine, and possesses high cationic reactivity. PEI “Max”-coated MNPs (PEI max-MNPs) are stable in deionized water, and positively charged. Thus, PEI max-MNPs electrostatically bind to plasmids. We attempted to introduce the green fluorescent protein (GFP) gene into a mouse embryonic carcinoma cell line, P19CL6 using PEI max-MNPs, and succeeded in establishing a highly efficient and low cytotoxic gene delivery system [107]. Furthermore, we applied this system to human fetal lung-derived fibroblasts (TIG-1 cells) using sixwell plates. Using MNPs, the transfected gene’s expression level increased 2-to 4-fold under optimum conditions (Figure 4, unpublished data). Furthermore, to assess whether the multiple plasmids were expressed in a single cell, we attempt to induce the expression of three fluorescent proteins GFP, cyan fluorescent protein (CFP), and yellow fluorescent protein (YFP). Most cells expressed these three proteins (Figure 5, unpublished data) indicating that gene delivery using MNPs could introduce and allow expression of multiple genes in a single cell.

## 5. Conclusions

The great promise of gene therapy for treating devastating, incurable diseases has yet to be realized. Less toxic and more efficient systems will be required, and robust research efforts in this regard are currently underway. Rapid advances have been made in adapting nanoparticle technology for basic biomedical and clinical research. Nanoparticles are already being used clinically to enhance MRI imaging, and drug delivery for cancer patients. Our own research has focused on gene delivery systems for autologous cell transplantation therapy, in which the patient’s own cells are transfected with the gene required to correct their condition. In particular, our laboratory and those of others have aimed to optimize magnetofection by developing better nanoparticle coating agents [38,40–51,53–55]. Nanoparticle size is another important parameter but there were few reports addressing this subject [111]. Since cells endocytose MNPs [51,100,101], MNP size has significant implications for transfection efficiency. PEI-MNPs forms magnetoplex, which increased its influence on the magnetic force. Furthermore, MNP size influences cytotoxicity [112], and more studies on this aspect of MNP technology will be crucial for enhancing transfection efficiencies.

The two research groups reported the important developments in the field of magnetofection. The first is the influence of the oscillating magnetic force on transfection [113,114]. The second is the use of MNP-heating, and -transfection [15,16]. The purpose of these studies have increased the efficiency of transfection, and/or induced a fever by oscillating MNPs for hyperthermia. The latter, a combination of MNP-heating and -transfection, was expected to research the efficacy of both hyperthermia and gene delivery. In the future, the studies of magnetofection using the oscillating MNPs could be developed as a novel methodology.

We found that PEI is an excellent cationic polymer for dispersing MNPs and that its water solubility, stability, and low toxicity contribute to enhancing transfection efficiency [95,115–119]. Derivation of iPSCs with the use of non-viral gene delivery using PEI max MNPs should provide a powerful tool for treating diseases such as Alzheimer’s, Huntington’s, and Parkinson’s by autologous cell transplantation. Reprogramming cells requires the action of multiple transcription factors. Our studies demonstrate that MNP-mediated transfection efficiently introduces at least three genes in a single cell. This indicates the feasibility of our system for one-step reprogramming.

## Figures and Tables

**Table 1:** Biomedical Applications of Magnetic Nanoparticles (MNPs).

	Purpose	References
**MRI**	Diagnosis	[1–8,57–61]
**DDS**	Anti-cancer therapy, Enzyme therapy	[9–11,22–31]
**Hyperthermia**	Anti-cancer therapy	[12–18,33–37]
**Gene Delivery**	Anti-cancer therapy, Cell transplantation therapy	[38–55]

**Table 2:** Gene delivery systems.

	Expression Type	Efficiency (%)	Cell Viability (%)	Safety
Virus*	Stable, or Transient	80–90%	80–90%	Low
Electroporation	Transient	50–70%	40–50%	High
TF reagent **	Transient	20–30%	80–90%	High

* Virus including adenovirus (transient), retrovirus (stable), and lentivirus (stable);

** TF reagent, transfection reagents including PEI (Polysciences Inc.), FuGENE HD (Promega), and Lipofectamine 2000 (Invitrogen);

All values are ours (unpublished experiments).

**Table 3:** Summary of magnetofection literature.

Author	Year	Vector	Magnetic Nanoparticles	Modifying Agent	Targeting Cell, or Tissue	TF Efficiency	Cell Viability (% of Control)	Reference
Kami D	2011	Plasmid	Iron oxide (γ-Fe_2_O_3_)	PEI max (MW: 25 k)	P19CL6	* 82%	100%	[107]

Pickard MR	2011	Plasmid	NeuroMag	-	Neural precursor cell	* 30%	70%	[39]

Hashimoto M	2011	Adenovirus, Biotin	SPION	PEI, Streptoavidin	HeLa	** 4-fold	-	[55]
		Adenovirus, Biotin	SPION	PEI, Streptoavidin	NIH3T3	** 10-fold	-	
		Adenovirus, Biotin	SPION	PEI, Streptoavidin	Mouse embryonic brain	-	-	

Biswas S	2011	Plasmid	Iron oxide (Fe_3_O_4_)	Aminooxy, Oxime ether	MCF-7	** 1425-fold	89%	[110]

B González	2011	Plasmid	SPION	Poly(propyleneimine) dendrimers	Saos-2 osteoblasts	* 12%	75%	[104]

Zhang H	2010	Plasmid	SPION	Branch PEI (MW: 25 k)	NIT3T3	* 64%	100%	[38]
		siRNA	SPION	Branch PEI (MW: 25 k)	NIT3T3	* 77%	100%	

Song HP	2010	Plasmid	PolyMag	Tat peptide	U251	* 60%	80%	[43]
		Plasmid	PolyMag	Tat peptide	Rat spinal cord	** 2-fold	-	

Arsianti M	2010	Plasmid	Iron oxide	Branch PEI (MW: 25 k)	BHK-21	-	60–90%	[51]

Shi Y	2010	Plasmid	Magnetite	Hyperbranch PEI (MW: 10 k)	COS-7	** 13-fold	-	[45]

Ang D	2010	Plasmid	Magnetite	Branch PEI (MW: 25 k)	COS-7	** 6-fold	70%	[46]

Tresilwised N	2010	Adenovirus	Iron oxide (Fe_2_O_3_, Fe_3_O_4_)	Branch PEI (MW: 25 k), Zonyl FSA fluorosurfactant	EPP85-181RDB	** 10-fold	-	[54]

Namgung R	2010	Plasmid	SPION	PEG, Branch PEI (MW: 25 k)	HUVEC	** 12-fold	80%	[48]

Yiu HH	2010	Plasmid	Iron oxide (Fe_3_O_4_)	PEI (MW: 25 k), MCM48 (Silica particle)	NCI-H292	** 4-fold	-	[49]

HC Wu	2010	Plasmid	Magnetite	Hydroxyapatite	Rat marrow stromal cells	* 60–70%	100%	[105]

Namiki Y	2009	Plasmid	Magnetite	Oleic acid, Phospholipid	HSC45	** 8-fold	-	[50]
		siRNA	Magnetite	Oleic acid, Phospholipid	Tissue sample from gastric cancer	-	-	

Kim TS	2009	Plasmid	PolyMag	-	Boar spermatozoa	-	-	[52]

Kievit FM	2009	Plasmid	SPION	PEI (MW: 25 k)	C6	* 90%	10%	[41]
		Plasmid	SPION	PEI (MW: 25 k), Chitosan	C6	* 45%	100%	
		Plasmid	PolyMag	-	C6	* 32%	66%	

Lee JH	2009	siRNA	MnMEIO	Serum albumin, PEG-RGD	MDA-MB-435-GFP	* 30%	-	[40]

Li Z	2009	Plasmid	Iron oxide	Poly-l-lysine	Lung tissue	*** 60%	-	[103]

Yang SY	2008	Plasmid	Iron oxide (Fe_3_O_4_)	Lipofectamine 2000	He99	-	-	[53]
		Plasmid	Iron oxide (Fe_3_O_4_)	DOTAP:DOPE	He99	-	-	

Pan X	2008	Plasmid	Magnetite	Oleic acid, Branch PEI (MW: 25 k), Transferrin	KB	** 300-fold	92%	[102]

Mykhaylyk O	2007	Plasmid	Iron oxide (Fe_2_O_3_, Fe_3_O_4_)	Branch PEI (MW: 25 k)	H441	* 49%	-	[42]
		Plasmid	Iron oxide (Fe_2_O_3_, Fe_3_O_4_)	Pluronic F-127	H441	* 37%	-	
		Plasmid	Iron oxide (Fe_2_O_3_, Fe_3_O_4_)	Lauroyl sarcosinate	H441	-	-	
		Plasmid	Iron oxide (Fe_2_O_3_, Fe_3_O_4_)	Branch PEI (MW: 25 k), Lauroyl sarcosinate	H441	-	-	

Morishita N	2005	Plasmid	Iron oxide (γFe_2_O_3_)	HVJ-E, protamine sulfate	BHK-21	** 4-fold	-	[47]
		Plasmid	Iron oxide (γ-Fe_2_O_3_)	HVJ-E, heparin sulfate	Liver, BALB/c mice (8 weeks age)	** 3-fold	-	

Scherer F	2002	Plasmid	SPION	PEI (MW: 800 k)	NIH3T3	** 5-fold	-	[44]
		Adenovirus	SPION	PEI (MW: 800 k)	K562	** 100-fold	-	
		Retrovirus	SPION	PEI (MW: 800 k)	NIH3T3	* 20%	-	

Mah C	2002	Adenovirus	Avidinylated magnetite	Biotunylated heparan sulfate	C12S	* 75%	-	[56]
		Adenovirus	Avidinylated magnetite	Biotunylated heparan sulfate	Adult 129/SvJ mice	-	-	

* indicates % of fluorescent positive cells analyzed by flow cytometric analysis.

** indicates analysis by luciferase activity assay compared with control. Transfection efficiency was indicated optimal transfection condition.

*** indicates transfection without magnetic force.

PEI: Polyethylenimine; PEI max: Deacaylated PEI; MNP: Magnetic nanoparticle; SPION: Superparamagnetic iron oxide nanoparticle; MW: Molecular weight; TF: transfection; PolyMag: Commercial Magnetofection reagent), NeuroMag (Commercial Magnetofection reagent); HVJ-E: hemagglutinating virus of Japan-envelope; DOTAP: 1,2-dioleoyl- 3-trimethylammonium-propane; DOPE: 1,2-dioleoyl-3-sn- phosphatidyl-ethanolamine; Tat peptide: cationic cell penetrating peptide; MeMEIO: Manganese-doped magnetism-engineered iron oxide; PEG: polyethylene glycol, Zonyl FSA fluorosurfactant: Lithium 3-[2-(perfluoroalkyl)ethylthio]propionate).
